# Mitochondrial Transfer as a Novel Therapeutic Approach in Disease Diagnosis and Treatment

**DOI:** 10.3390/ijms24108848

**Published:** 2023-05-16

**Authors:** Vicente Javier Clemente-Suárez, Alexandra Martín-Rodríguez, Rodrigo Yáñez-Sepúlveda, José Francisco Tornero-Aguilera

**Affiliations:** 1 Faculty of Sports Sciences, Universidad Europea de Madrid, Tajo Street, s/n, 28670 Madrid, Spain; 2 Faculty of Education and Social Sciences, Universidad Andres Bello, Viña del Mar 2520000, Chile

**Keywords:** mitochondrial transfer, disease diagnosis, disease treatment, cell death, neurodegenerative diseases, metabolic disorders, cancer

## Abstract

Mitochondrial dysfunction is a hallmark of numerous diseases, including neurodegenerative disorders, metabolic disorders, and cancer. Mitochondrial transfer, the transfer of mitochondria from one cell to another, has recently emerged as a potential therapeutic approach for restoring mitochondrial function in diseased cells. In this review, we summarize the current understanding of mitochondrial transfer, including its mechanisms, potential therapeutic applications, and impact on cell death pathways. We also discuss the future directions and challenges in the field of mitochondrial transfer as a novel therapeutic approach in disease diagnosis and treatment.

## 1. Introduction

Mitochondria are organelles within cells that play a vital role in energy production and metabolism. Mitochondrial dysfunction has been implicated in a wide range of diseases, including neurodegenerative disorders, metabolic syndromes, and cardiovascular disease [1]. In recent years, there has been increasing interest in developing novel therapeutic approaches to target mitochondrial dysfunction and prevent or treat these diseases.

Mitochondrial dysfunction is a common feature of many diseases, including neurodegenerative disorders, metabolic disorders, and cancer. This dysfunction can lead to impaired energy metabolism, increased oxidative stress, and altered calcium homeostasis, ultimately contributing to disease pathogenesis [2,3,4]. Mitochondrial-targeted therapies have emerged as a promising strategy for treating these diseases by restoring mitochondrial function and reducing oxidative stress [5]. However, limitations in drug delivery and specificity have hindered the success of these therapies [6]. Therefore, there is a critical need for novel therapeutic approaches that can effectively target mitochondrial dysfunction. 

Researchers in biology and medicine have recently shown a great deal of interest and attention in the phenomena of mitochondrial transfer. Different mechanisms, such as gap junction channels (GJCs), extracellular vesicles (EVs), and tunneling nanotubes (TNTs), are used for intercellular mitochondrial transport. In 2004, firstly Rustom et al. observed TNTs in cultured rat pheochromocytoma PC12 cells, human embryonic kidney (HEK) cells, and normal rat kidney cells [7]. Moreover, TNTs were later discovered by Koyanagi et al. to be capable of transferring mitochondria between neonatal rat cardiomyocytes and endothelial progenitor cells [8]. These structures have been seen in nerve, muscle, and cancer cells as well as immune cells [9]. These two investigations could be considered as pioneering in this field.

As therapy, the first mitochondrial transfer experiment was conducted in 2006 and showed that mitochondria from bone marrow stromal cells (BMSCs) could move to mitochondria-deficient A549 lung cancer cells and restore their aerobic respiration, but not release mitochondria or mtDNA from the media [10]. In this research, authors investigate whether the transfer of functional mitochondria or mtDNA can restore mitochondrial function to damaged cells in stem/progenitor cells or other somatic cells. A549 cells were employed, which were pretreated with ethidium bromide to cause mtDNA mutation and depletion. As a result, the cells were unable to grow or respire aerobically, with the exception of when they were placed in a permissive medium that contained uridine and pyruvate to support anerobic glycolysis [10]. However, adult nonhematopoietic stem/progenitor cells from human bone marrow (hMSCs) or skin fibroblasts were cocultured with the A549° cells. The cocultures generated copies of functionally restored A549° cells. Thus, it was demonstrated that mitochondria are more active than previously thought, with the ability to travel across cells or carry mtDNA. In mammalian cells with dysfunctional mitochondria, active transfer from adult stem cells to somatic cells can restore aerobic respiration [10]. Therefore, the transfer of mitochondria from donor cells to recipient cells appears to be a promising approach to realize intercellular energy synchronization, as mounting evidence of mitochondrial transfer between cells has shown that mitochondria are much more active than previously understood [11,12].

In this regard, recent research has highlighted the importance of mitochondrial transfer in maintaining mitochondrial function and preventing cell death in disease; therefore, its future importance in pathological processes has been recognized. Mitochondrial transfer involves the exchange of mitochondrial components, such as DNA, RNA, and proteins, between cells and has been shown to improve mitochondrial function and reduce oxidative stress in various cell types [1,13]. Several studies have suggested that mitochondrial transfer may be a promising therapeutic approach for treating diseases associated with mitochondrial dysfunction. For example, a recent study demonstrated that mitochondrial transfer could rescue neurons from cell death in a mouse model of Parkinson’s disease [14]. Similarly, another study showed that mitochondrial transfer could improve glucose metabolism in diabetic mice by restoring mitochondrial function in pancreatic beta cells [14].

One example of a disease in which mitochondrial dysfunction plays a key role is Alzheimer’s disease (AD). Studies have shown that mitochondrial dysfunction, particularly impaired mitochondrial respiration and decreased adenosine triphosphate (ATP) production, is an early event in the development of AD. Mitochondrial dysfunction can lead to increased oxidative stress, which can damage cells and contribute to the pathogenesis of AD [15]. In addition, mitochondrial dysfunction has been linked to the accumulation of amyloid-beta and tau proteins, which are characteristic features of AD pathology [16]. Targeting mitochondrial dysfunction may therefore represent a promising strategy for preventing or treating AD.

Another disease in which mitochondrial dysfunction is implicated is type 2 diabetes mellitus (T2DM). Mitochondrial dysfunction in T2DM is characterized by decreased mitochondrial content and impaired mitochondrial function, which can contribute to insulin resistance and impaired glucose metabolism [17]. Additionally, mitochondrial dysfunction can lead to increased oxidative stress, inflammation, and apoptosis, which have all been linked to the development of T2DM [18]. Developing therapies that target mitochondrial dysfunction may therefore be a promising approach for treating T2DM.

Several approaches have been proposed for targeting mitochondrial dysfunction in disease. One approach is to target the mitochondrial respiratory chain, which is responsible for producing ATP. This can be achieved using electron transport chain (ETC) modulators, which can improve mitochondrial respiration and ATP production (Bhatti et al., 2017) [19]. Another approach is to target mitochondrial biogenesis, which involves the generation of new mitochondria. This can be achieved through the use of activators of the peroxisome-proliferator-activated receptor gamma coactivator-1 alpha (PGC-1α), a key regulator of mitochondrial biogenesis [20]. Other approaches include the use of antioxidants to reduce oxidative stress and the use of mitophagy inducers to promote the clearance of damaged mitochondria [21].

In the present review, we discussed the actual knowledge in this novel area. Mitochondrial dysfunction is implicated in a wide range of diseases, and developing novel therapeutic approaches to target mitochondrial dysfunction emerges as a promising strategy for preventing or treating these diseases. Targeting the mitochondrial respiratory chain and mitochondrial biogenesis, as well as reducing oxidative stress and promoting mitophagy, may represent promising therapeutic approaches. However, further research is needed to optimize mitochondrial transfer as a therapeutic strategy, including identifying the optimal cell sources for transfer, improving the efficiency of transfer, and minimizing potential risks, such as the transfer of disease-causing mitochondrial mutations [22].

## 2. Overview of Mitochondrial Transfer and Its Mechanisms

Mitochondrial transfer is a process by which mitochondria can be transferred from one cell to another [23]. This process has garnered significant interest in recent years due to its potential applications in the treatment of mitochondrial disorders, aging-related diseases, and other conditions [10]. In this discussion, we will provide an overview of mitochondrial transfer, including its mechanisms, potential benefits, and limitations. Mitochondrial transfer can occur through several mechanisms, including cell-to-cell fusion, exosome-mediated transfer, and tunneling nanotubes [24]. Cell-to-cell fusion involves the fusion of two cells, resulting in the exchange of cellular components, including mitochondria. This process has been observed in a variety of cell types, including immune cells and cancer cells. In some cases, it occurs spontaneously, while in others, it can be induced by certain stimuli, such as viral infection or exposure to chemicals [25]. The exact mechanisms of cell-to-cell fusion are still not fully understood, but it is thought to involve the interaction of proteins on the cell surface, as well as the rearrangement of the cytoskeleton to facilitate the fusion of the plasma membranes.

For a better understanding, it is relevant to point out that for a very long time, it was believed that mitochondria were restricted to the cytoplasm. They do, in fact, frequently change their programming and move inside their cells [26]. For its enormous impact on mitochondrial homeostasis in neurons, the bidirectional (anterograde and retrograde) internal axonal transport of mitochondria has been extensively studied [27]. However, there is currently a lot of interest in the crucial roles that mitochondrial transfer plays in tissue homeostasis and development [24]. Regarding mechanisms by which donor cells initiate the export of mitochondria and how these mitochondria are packaged and transported to recipient cells, Spees et al. demonstrated the processes by which the intercellular transfer of healthy mitochondria from mesenchymal stem cells (MSCs) to mammalian cells with malfunctioning mitochondria happens in 2006 [10]. Mitochondria encode a number of their own proteins, and translation occurs along the inner membrane of the mitochondrial matrix. Concretely in this study, the possibility of vesicular transfer was substantiated by the observation that MSCs deposited vesicles in culture dishes containing mitochondria, as indicated by the presence of mitochondria-targeted red fluorescent protein. However, the authors were unable to demonstrate the transfer of mitochondria from isolated or cocultured platelets. Therefore, they conclude that mitochondrial transfer appeared to entail an active cellular process as opposed to the passive uptake of cellular fragments or organelles.

Moreover, as previously mentioned, Rustom et al. originally discovered the movement of organelles across mammalian cells via TNTs in 2004 [7]. Since then, growing evidence of mitochondrial transport between cells has shown that mitochondria are significantly more active than previously believed and it seems like a promising strategy to achieve intercellular energy synchronization which involves the transfer of mitochondria from donor cells to recipient cells [28]. Notably, spontaneous mitochondrial transfer between cells also occurs under physiological conditions during tissue homeostasis and development, which expands our understanding of mitochondrial transfer. Under pathological conditions, however, the intercellular mitochondrial transfer appears to not only rescue tissue damage, which has been reported frequently in the central nervous system (CNS), cardiovascular system, and respiratory system, but also to contribute to multifunctional cellular activity and has an effect on tumor therapy resistance and inflammation regulation [28].

One of the potential benefits of cell-to-cell fusion as a mechanism of mitochondrial transfer is that it allows for the transfer of intact, functional mitochondria. Mitochondria that are transferred via other mechanisms, such as exosome-mediated transfer or tunneling nanotubes, may be damaged or dysfunctional due to the stresses of the transfer process [9]. Additionally, cell-to-cell fusion allows for the transfer of other cellular components, such as organelles, enzymes, and proteins, which may be beneficial in certain contexts [29]. Despite its potential benefits, cell-to-cell fusion also has several limitations. One of the major concerns is the potential for the transfer of genetic material, particularly in the context of cancer cells. The fusion of cancer cells with healthy cells can result in the transfer of oncogenes or other cancer-promoting genetic material, which can contribute to the spread of cancer [30]. Additionally, the use of cell-to-cell fusion as a method of mitochondrial transfer is still in the early stages of research, and further studies are needed to fully understand its potential and limitations.

In this line, exosome-mediated transfer is another mechanism by which mitochondria can be transferred from one cell to another [31]. Exosomes are small, membrane-bound vesicles that are secreted by cells and can contain a variety of cellular components, including RNA, DNA, and proteins [32]. Recent studies have shown that exosomes can also contain intact, functional mitochondria, which can be transferred to other cells. This process has been observed in various cell types, including mesenchymal stem cells and neurons. Tunneling nanotubes are thin, membrane-bound structures that allow for direct communication and exchange of cellular components, including mitochondria, between cells. This process has been observed in various cell types, including immune cells and cancer cells [33].

Exosome-mediated transfer of mitochondria is thought to occur via several mechanisms. One possibility is that the exosomes are taken up by the recipient cells, either via endocytosis or by direct fusion with the plasma membrane. Once inside the recipient cell, the exosomes can release their contents, including mitochondria, which can then be integrated into the recipient cell’s metabolic machinery [34]. One potential advantage of the exosome-mediated transfer of mitochondria is that it can occur over long distances, allowing for the transfer of mitochondria between cells that may not be in direct contact with each other [31]. Additionally, exosomes can provide a protective environment for the transferred mitochondria, shielding them from the stresses of the extracellular environment [31].

However, the exosome-mediated transfer of mitochondria also has several limitations. One of the major concerns is the potential for the transfer of damaged or dysfunctional mitochondria. Exosomes that are secreted by cells under stress or in diseased states may contain mitochondria that are damaged or dysfunctional, which could negatively impact the recipient cell’s metabolism [35]. Additionally, the transfer of mitochondria via exosomes may not be as efficient as other mechanisms, such as the cell-to-cell fusion [31].

Mitochondrial transfer has several potential benefits, particularly in the treatment of mitochondrial disorders. Mitochondrial disorders are a group of genetic disorders that result from mutations in mitochondrial DNA or nuclear DNA that affect mitochondrial function [36]. These disorders can cause a wide range of symptoms, including muscle weakness, developmental delays, and cognitive impairment. Mitochondrial transfer has been proposed as a potential treatment for these disorders, as it can provide healthy mitochondria to cells that are affected by mitochondrial dysfunction. This approach has shown promise in preclinical studies, particularly in the context of oocyte transfer and stem cell therapy [37].

Despite its potential benefits, mitochondrial transfer also has several limitations. One of the major concerns is the potential for immune rejection of the transferred mitochondria, as these mitochondria contain genetic material from a different individual [38]. This concern has been raised in the context of oocyte transfer, as well as stem cell therapy. Additionally, there is a risk of introducing mitochondrial mutations or heteroplasmy, which can lead to unpredictable outcomes. Further research is needed to address these concerns and develop safe and effective mitochondrial transfer approaches [39].

Mitochondrial transfer is a process by which mitochondria can be transferred from one cell to another. This process has several potential benefits, particularly in the treatment of mitochondrial disorders, but also has limitations and potential risks. Further research is needed to fully understand the mechanisms of mitochondrial transfer and develop safe and effective approaches for its clinical use.

## 3. Methodology

In this study, we utilized a comprehensive literature search methodology to gather relevant information. Both primary and secondary sources were searched, including scientific articles, bibliographic indexes, and databases, such as PubMed, Scopus, Embase, Science Direct, Sports Discuss, ResearchGate, and the Web of Science. To ensure a systematic approach, MeSH-compliant keywords related to mitochondrial transfer, neurodegenerative disorders, metabolic disorders, death pathways, cancer, drug resistance, and regenerative medicine were used to search for articles published from 1 April 2003 to 1 March 2023.

To establish the inclusion criteria, a team of six review authors thoroughly screened the titles and abstracts of all retrieved manuscripts. Exclusion criteria were then applied to studies that utilized outdated data beyond the proposed timeline, had irrelevant topics that did not align with the study’s focused purpose, or were not written in English. The same team of nine review authors who conducted the study selection independently extracted information from the selected studies. Results were then discussed among the team to produce the present narrative review, ensuring a robust and comprehensive approach to synthesizing the available evidence.

## 4. Methods for Measuring Mitochondrial Transfer In Vitro and In Vivo

Mitochondrial transfer is a critical cellular mechanism that allows cells to maintain healthy mitochondria and sustain proper cellular function. Measuring mitochondrial transfer in vitro and in vivo is essential to understand the underlying mechanisms of this process and its potential implications in the health and disease [40]. The capacity to examine mitochondrial function in the presence of intact circulatory and regulatory systems is a strength of these techniques. Ex vivo experiments allow a reductionist approach to probe mitochondrial function at specific molecular and cellular levels, information that is critical to understand mechanisms by which mitochondrial function changes with aging and disease [41], as well as pathways by which exercise and pharmacological interventions have beneficial effects. Furthermore, the ability to quantify mitochondrial DNA copy numbers, expression (mRNA and protein) of various mitochondrial proteins, mitochondrial enzyme activities, and posttranslational protein modifications allows for the collection of a massive amount of complementary data from the same biopsy sample used for mitochondrial function measurements [42,43]. These approaches allow for a thorough analysis of mitochondrial function as well as the many molecular and cellular pathways that underpin mitochondrial alterations associated with aging, disease, and even physical activity [42].

Regarding mitochondrial isolation procedures, Cogswell et al. discovered two different mitochondrial populations in skeletal muscle [44]. Subsarcolemmal mitochondria (20% of total mitochondrial content) are described as those located within 2 microns of the sarcolemma, are frequently densely grouped near nuclei, and are quickly freed by gentle mechanical homogenization. Intermyofibrillar mitochondria (80% of total mitochondria) are embedded within the contractile apparatus and are best freed by softening the tissue prior to homogenization with proteolytic enzymes [45]. Improper proteolytic enzyme use may cause mitochondrial damage; however, precise regulation of proteolytic action prevents organelle damage while efficiently releasing the organelles from contractile machinery. Once homogenization of the tissue has taken place, it should be considered that mitochondria are tiny in comparison to other components of the muscle homogenate, and they can be separated using differential centrifugation. At low speeds, myofibrillar components form a pellet, whereas sedimentation of mitochondria requires more centrifugal force. Laboratory investigations have demonstrated that after isolation, the function of isolated mitochondria stays unaltered for up to 18 h when stored at 4 °C [46]. When evaluating the function of isolated mitochondria, mitochondrial integrity is a key factor to examine. Regarding this process, during homogenization operations, the outer membrane is easily broken, causing cytochrome c to exit into the buffer and become rate-limiting to oxygen consumption and ATP production [46]. Citrate synthase activity in isolated mitochondria can be measured before and after membrane disruption by freeze–thaw cycles and enzyme extraction with Triton X-100 to determine mitochondrial integrity. The activity of citrate synthase is determined using standard spectrophotometric techniques [47]. Given the possibility of complications, the potential for toxicity must be taken into account. For instance, the progression of Parkinson’s disease is associated with the accumulation of pathological α-synuclein (α-syn) [48]. Recent evidence suggests that pathological α-syn aggregates can bind with high affinity to mitochondria, resulting in toxicity and mitochondrial dysfunction [28].

In vitro methods provide a controlled and reproducible environment to study mitochondrial transfer. One of the advantages of using fluorescent dyes is that they allow for real-time monitoring of mitochondrial transfer from donor to recipient cells. For instance, the MitoTracker dyes are frequently used for staining mitochondria in donor cells, which allows for the tracking of the transfer of mitochondria to recipient cells by flow cytometry or confocal microscopy [11,40]. The labeling of mitochondria with fluorescent dyes also provides a reliable and quantitative method to measure the efficiency of mitochondrial transfer between different cell types.

In vitro studies are performed in a controlled environment outside of the living organism, usually using cultured cells. One of the most common methods for measuring mitochondrial transfer in vitro is using fluorescent dyes [49]. The dyes are loaded into donor cells, which are then co-cultured with recipient cells. The transfer of mitochondria can be visualized by fluorescence microscopy, and the amount of transfer can be quantified using a flow cytometry [10]. Another in vitro method for measuring mitochondrial transfer is the use of mitochondrial DNA (mtDNA) sequencing. By sequencing mtDNA from donor and recipient cells, the presence of donor mtDNA in recipient cells can be identified, indicating the transfer of mitochondria [50]. The mtDNA sequencing method has the advantage of being able to detect the transfer of functional mitochondria, which can result in improved cellular metabolism and bioenergetics.

However, there are limitations to in vitro methods for measuring mitochondrial transfer. In vitro systems may not fully represent the complexity of in vivo environments, and the experimental conditions may not accurately reflect the physiological and metabolic state of cells in vivo [51]. Additionally, the use of fluorescent dyes and mtDNA sequencing methods may require invasive procedures to load donor cells, which could affect cell viability and induce cellular stress responses [52].

In contrast, in vivo research is conducted within a living organism. In vivo mitochondrial transfer measurements are more challenging than in vitro measurements due to the physiological complexity of working with organisms that are alive. In vivo methods for measuring mitochondrial transfer provide a more physiologically relevant context for studying the transfer of mitochondria between cells. One common approach is to use animal models, such as mice or rats, to investigate the transfer of mitochondria between tissues or organs. For example, the transfer of mitochondria from mesenchymal stem cells to lung tissue has been studied in mouse models of acute lung injury [52].

As performed in vitro, mitochondrial labeling is a method for measuring mitochondrial transfer in vivo. Likewise, another method used in both is the use of mtDNA sequencing [34]. However, a particular in vivo approach for measuring mitochondrial transfer is using mitochondrial DNA (mtDNA) haplotypes. mtDNA haplotypes can be used to track the transfer of mitochondria from maternal sources, which can be particularly useful in the study of maternal–fetal mitochondrial transfer during pregnancy [39]. This approach can provide insight into the role of maternal–fetal mitochondrial transfer in fetal development and disease. Specifically, mitochondrial DNA haplotypes are regions of mitochondrial DNA that aggregate with other mitochondrial sequences to indicate the phylogenetic origins of maternal lineages. Haplotypes of mitochondrial DNA are associated with a variety of phenotypes and diseases [53]. Changes in mtDNA copy number can modulate chromosomal gene expression patterns and promote differentiation in tumor cells [54]. mtDNA haplotypes can similarly affect chromosomal gene expression patterns in stem cells [55] and cancers [56]. Specific regions of mtDNA that designate the phylogenetic origins of maternal lineages [57]. In this regard, mtDNA haplotypes are associated with adaptation to warm and frigid environments, susceptibility to age-related diseases such as cancer, diabetes, Alzheimer’s, and Parkinson’s, and fertility in a variety of species [58]. Additionally, based on adult mice’s studies, it has been demonstrated that mtDNA segregation resulting from donor–recipient mtDNA mismatch in mitochondria replacement therapy is associated with undetermined risks. Pan et al. specified that mitochondrial segregation inevitably occurs in offspring from mitochondrial replacement manipulation if no haplotype matching has been conducted [59]. This study indicates that genetic similarity between donor and recipient mtDNA has the potential to circumvent the segregation bias toward pathogenic maternal mtDNA in tissues of mitochondria replacement offspring. These results recommended that mtDNA haplotype matching should be undertaken between the donor and recipient, as it could “fool” the nucleus into treating the donated mtDNA and the native pathogenic mtDNA the same, thereby eliminating any proliferative advantage, and circumvent any segregation bias and prevent the onset of mitochondrial diseases [59].

All the methods discussed above are relevant for researching the mechanism of cell function restoration or improvement following mitochondrial transfer. However, they are currently unable to meet the cell therapy industry’s requirement for a huge quantity of mitochondria-transplanted cells [60]. The coculture technology has significant advantages because it is safe and has a high throughput, but its low efficiency and heterogeneity remain significant constraints. For instance, droplet microfluidics is a method that disperses a continuous flow of chemical reagents, cells, or other biomaterials into discrete micrometer-sized quantities known as droplets [61]. The closed microenvironment of droplets reduced the travelling distance of isolated mitochondria and improved their likelihood of contacting the cell, allowing mitochondria uptake by the cell and enhancing mitochondria transfer efficiency [62]. Furthermore, because isolated mitochondria are smaller in size than droplets (1 to 40 m in diameter), the isolated mitochondria were equally enclosed in each droplet. The concentration of the isolated mitochondria suspension could be adjusted to control the number of isolated mitochondria encapsulated in droplets. Sun et al. reported that the mitochondrial transfer efficiency slightly increased from 73 to 78%, with the concentration increasing from 0.25 to 1.0 U [61]. In this regard, exogenous mitochondrial transplantation has recently been studied in CNS disease or injury, and studies reported an amount of mitochondria suspended in vesicles for a total of 50, 100, or 150 μg [63]. In addition to animal models, advances in imaging techniques have also enabled the visualization of mitochondrial transfer in vivo. For example, two-photon microscopy can be used to track the transfer of mitochondria between cells in live animals [64]. This technique allows for the visualization of mitochondrial dynamics and the transfer between cells in real time, which can provide valuable information about the mechanisms underlying mitochondrial transfer in vivo.

Each method for measuring mitochondrial transfer has its strengths and limitations. In vitro, methods allow for precise control of experimental conditions, and fluorescent dyes provide a straightforward method for visualizing and quantifying mitochondrial transfer. However, these methods do not fully represent the complexity of in vivo environments. In vivo methods are more challenging to perform, but they provide a more realistic representation of mitochondrial transfer in living organisms. Mitochondrial labeling allows for the tracking of mitochondria within the organism, while mtDNA sequencing provides direct evidence of mitochondrial transfer. However, these methods may not accurately represent the transfer of mitochondria in specific cell types or organs of interest.

Measuring mitochondrial transfer is essential to understand the underlying mechanisms of this process and its implications in health and disease. In vitro methods such as fluorescent dyes and mtDNA sequencing generate a straightforward and precise method for measuring mitochondrial transfer. In vivo methods such as mitochondrial labeling and mtDNA sequencing provide a more realistic representation of mitochondrial transfer in living organisms. Each method has its strengths and limitations and should be selected based on the research question and experimental design. Examples of animal (Table 1) and human (Table 2) trials made possible by the techniques discussed in this article are provided below.

## 5. Therapeutic Potential of Mitochondrial Transfer in Neurodegenerative Diseases

Changes in the integrity and activity of mitochondria can contribute to synaptic injury and neuronal mortality, particularly in degenerative diseases associated with aging [79]. Mitochondria play a critical role in metabolic balance in all multicellular eukaryotes. ATP produced by mitochondria is essential in the nervous system to provide proper electrochemical gradients and reliable synaptic transmission. Notably, various mitochondrial abnormalities have been linked to neurological diseases. Membrane leakage and electrolyte imbalances, activation of pro-apoptotic pathways, and mitophagy are among the processes implicated in the development of neurodegenerative illnesses, such as Alzheimer’s, Parkinson’s, and Huntington’s disease, as well as ischemic stroke. Specifically, in AD, the mitochondrion does not function properly, jeopardizing some fundamental processes of the nerve cell. Importantly, mitochondrial dysfunctions occur not only in the early phases of AD but also prior to the onset of clinical symptoms. Various experimental models of the disease demonstrate an altered function of mitochondria, which play a crucial role in initiating degenerative processes by causing extensive oxidative damage to neuronal lipids, nucleic acids, and proteins in the brains of AD patients [19]. In this regard, the transfer of mitochondria is a physiological process that occurs naturally. Neurons can release damaged mitochondria for disposal and recycling by transferring them to the astrocytes [80]. The ability to exchange mitochondria may represent a potential mode of cell–cell communication in the CNS, and astrocytes may provide neurons with functional mitochondria. This exchange occurs via tunneling nanotubes, extracellular vesicles, or cell fusion [81]. Nevertheless, the precise mechanism by which exogenously injected mitochondria induce a beneficial effect remains unknown. Specifically, the contribution or necessity of the internalization of exogenous mitochondria into a relevant tissue target is not completely understood [82].

As the leading cause of dementia in the elderly, AD is a formidable socioeconomic and healthcare challenge in the 21st century. As dementia affects a large population, addressing individual risk factors could represent effective global prevention. It entails acting on elements that can be controlled in whole or in part, whereas age and genetics cannot be modified by definition [83]. Concretely, mitochondrial impairment of mitochondrial-specific autophagy, namely, mitophagy, has emerged as an essential component of the cellular processes that contribute to the development of Alzheimer’s disease pathologies, including amyloid-β plaques (A-β) and neurofibrillary tangles (NFTs) [84]. Despite significant progress in the understanding of the molecular basis of neurodegenerative diseases, numerous clinical trials have failed to identify pharmaceuticals that successfully delay or halt disease progression [85,86]. However, the translocation of mitochondria, the mitochondrial genome, or other mitochondrial components across cells has lately re-captured the scientific community’s interest [85]. Horizontal mitochondrial transfer describes the movement of mitochondria across cells and can occur under normal and pathological circumstances in mammalian cells either in vitro or in vivo [87]. To correct mitochondrial defects or, as in the case of tumor cells, to alter their functional abilities and responsiveness to treatment, mitochondrial transfer can supply an external mitochondrial source [88].

The amount of articles indicating that mitochondrial dysfunction has been identified as a potential crucial target of multiple scoliosis (MS) pathology has increased in recent years [89]. Multiple studies have demonstrated mitochondrial dysfunction in MS patients, which is correlated with axonal degeneration and disease progression. The number of mitochondria, microtubules, and axonal edema was drastically diminished in demyelinated spinal cord lesions, as determined by the ultrastructural analysis [90,91]. Several independent studies have demonstrated that molecular alterations in MS neurons converge on mitochondria. The mechanism that contributes most to the degeneration of demyelinated axons is an imbalance between the increased energy demand for nerve conduction and the production of ATP. In neurons, Na^+^/K^+^-ATPase, present in Ranvier’s nodes, creates impulse transmission. The action of Na^+^/K^+^-ATPase is amplified in demyelinated axons to maintain impulse conduction despite the increased consumption of ATP [92]. When ATP is in short supply, excessive sodium concentration in the axon causes the Na^+^/Ca^2+^ exchanger to operate in reverse, resulting in calcium overload, activation of proteases, and degeneration of the demyelinated axon [93]. However, and relatively recently, the ability to transfer healthy mitochondria from one cell to another has become an attractive therapeutic strategy [94]. Mitochondrial transplantation indicates that the transfer of live mitochondria into damaged cells can treat a variety of diseases, including neurodegenerative disorders [95,96].

In animal models, mitochondrial transfer has been used to treat neurodegenerative illness and other central nervous system (CNS) disorders, in addition to cardiovascular concerns [97]. Because of the variability of pathophysiology and the efficiency of mitochondrial internalization into damaged tissues, the success of mitochondrial treatment is likely to vary between individuals [98]. The volume and quality of mitochondria, as well as the precise pathways of organelle distribution, are critical for successful mitochondrial absorption by target tissues [99]. Mitochondria removed from the patient might be sent to the CNS via several methods [94,100]. Intracerebral injection can be used for therapeutic purposes; however, it is an invasive procedure. Intrathecal injection is a fluid-phase delivery route to the CNS that involves delivering materials to the intrathecal region surrounding the spinal cord [94]. Intrathecal administration is less painful for patients and allows for a greater amount of therapeutic ingredients than intracranial injection. For instance, some studies specified that in vivo intrathecal sustained administration of FM19G11 in rats with spinal cord injury resulted in a greater number of neurofilament TUJ1-positive fibers crossing the injured area and an increase in neural precursor Vimentin-positive cells [101]. Overall, FM19G11 exerts a significant effect on the self-renewal of ependymal stem progenitor cells with a plausible neuroprotective function, providing functional benefits for the treatment of spinal cord injury [101]. 

**Figure 1:**
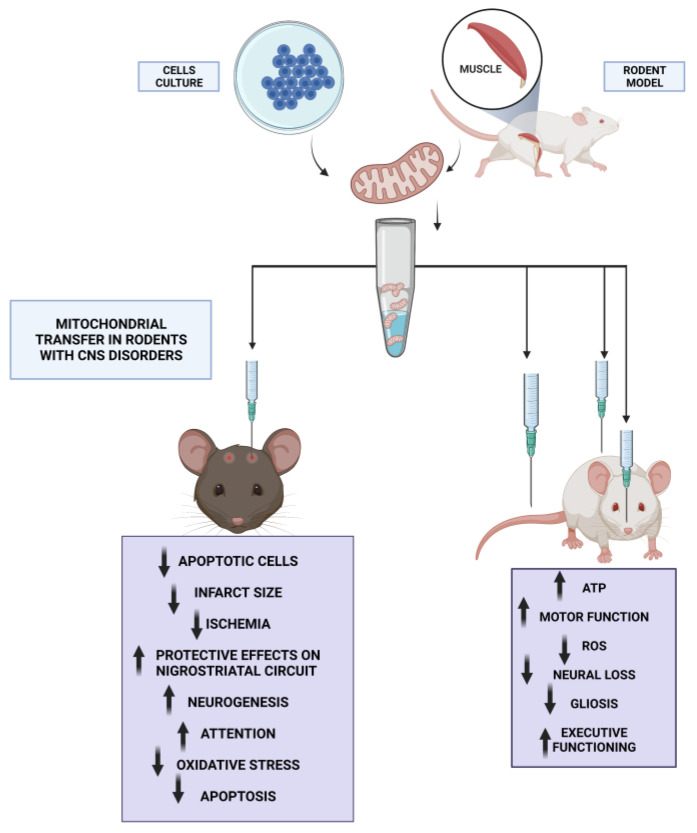
Main advances obtained after mitochondrial transfer in animals with central nervous system disorders. Nasal, intravenous, intracerebroventricular, and intrathecal injections are shown.

The cardiovascular system is another area that has seen this sort of interventions [76]. In this regard, Masuzawa et al. (2013) reported the transplantation of isolated autologous mitochondria into ischemic heart tissue in vivo with positive results. Immediately after injections, mechanical function is enhanced. Benefits can be visible within 10 min [102]. Regarding CNS, both in situ and systemic methods have been employed to introduce mitochondrial transplantation (MT) into the brain in vivo. Injured rats had labeled mitochondria inserted into their spinal cords using stereotactic surgery, and 24 h later, the transplanted mitochondria were discovered within the microglia [63]. Moreover, in a 6-hydroxydopamine (6-OHDA) rat model of Parkinson’s disease (PD), transplanted mitochondria labeled with an allogenic peptide were injected specifically into the medial forebrain bundle. Chan et al. discovered that MT elicited protective effects on nigrostriatal circuit neurons and that following a three-month observation period, the motor function of PD rodents improved, mitochondrial function increased, and the cytotoxic effects of 6-OHDA diminished [68]. Another study found that injecting hamster-isolated mitochondria into the brains of ischemia rats improved motor function. In relation to this, Huang et al. found that brain tissue from MT-treated rats showed a considerable reduction in apoptotic cells and infarct size, indicating that MT protects neurons in the aftermath of ischemia (Figure 1) [103]. Both intravenous injection and sonography-guided catheter administration of mitochondria through the carotid system have been studied as potential methods of transporting mitochondria to the brain [104]. Mice with MPTP-induced Parkinson’s disease show higher ATP content and enhanced locomotor activity 2 h after intravenous administration, indicating that mitochondria have spread throughout the body (Figure 1) [64]. In accordance with the study, Shi et al. found that mitochondrial transplantation enhanced motor function, decreased reactive oxidative species (ROS) production, and restored ATP levels and complex I activity [105].

Targeting mitochondrial deficits is extremely challenging due to the multiple, and even contradictory, modes of mitochondrial dysfunction in the pathogenesis of Alzheimer’s disease and their complex regulation. The experimental strategy of Nitzan et al. (2019) aimed to overcome this limitation by employing active and functional mitochondria, thereby allowing mitochondria to function as whole organelles as opposed to focusing on only one of their dysfunctional functions [70]. The results of their analysis indicate that the transfer of functionally active mitochondria, designed to replicate mitochondrial function efficiently, is advantageous for treating AD deficits, as it corrects cognitive deficits, brain pathology, and mitochondrial defects in an AD mouse model [70]. Specifically, AD mice (amyloid injected intracerebroventricular) were treated with isolated human mitochondria by intravenous (IV) in this new in vivo investigation to examine the effect of transplanting active, intact mitochondria. In addition, citrate synthase and cytochrome c oxidase activities were measured to be comparable to those of untreated control mice in mitochondria-treated AD mice [70]. In summary, AD mice treated with exogenous mitochondria exhibited cognitive improvements comparable to untreated control mice 14 days after mitochondrial transplantation. In addition, in the hippocampus of treated mice as compared to untreated AD mice, neuronal loss and gliosis were significantly reduced (Figure 1). 

Proceeding with other advantages of mitochondrial transplantation into the nervous system, experiments injected mitochondria transgenically labeled with a green fluorescent protein (GFP) from cellular cultures or limb muscle into the damaged spinal cord of rats. Gollihue et al. (2017) [63,106] found that mitochondria transplanted into microglial cells and spinal cord motoneurons produced a short-term neuroprotective effect. Moreover, Kuo et al. reported that transplanting mitochondria into injured sciatic nerves affected the maintenance of cytoskeletal integrity and enhanced nerve-conducting properties and animal behavior [107]. In an in vitro model of oxygen–glucose deprivation, it was discovered that conditioned media derived from endothelial progenitor cells contain functional mitochondria that are efficiently incorporated into brain endothelial cells, thereby reducing the permeability of these brain cells and promoting angiogenesis. Interestingly, an increase in ATP, mtDNA, and TOM40 protein was associated with the protective effect of mitochondria derived from endothelial progenitor cells [12]. In addition, mitochondria transported from transplanted stem cells promoted angiogenesis, lowered infarct volume, and enhanced functional recovery in a model of ischemic stroke, as reported by Liu et al. (2019) [108].

Regarding ischemic stroke, autologous mitochondrial transplantation from skeletal muscle has been successfully achieved by Zhang et al. in the lateral ventricles of rat brains (Figure 1) [109]. In terms of global mortality and disability, ischemic stroke is second only to heart disease. The brain is especially vulnerable to the long-term decrease in oxygen and glucose delivery caused by arterial thrombosis or embolism because of its high intrinsic metabolic activity [110]. Using a model of middle cerebral artery (MCA) blockage, they replaced dysfunctional mitochondria with healthy ones following an ischemic stroke. An intracerebroventricular MT was shown to lower cellular oxidative stress and apoptosis, reduce brain infarct volume, restore neurological impairments, suppress reactive astrogliosis, and stimulate neurogenesis (Figure 1) [109]. They found that improved neurological outcomes were linked to a larger number of viable mitochondria in the cerebrospinal fluid [109]. Similarly, treatment with exogenous mitochondria mitigated brain edema and blood–brain barrier (BBB) permeability in rodents with traumatic brain injury [111]. In relation to this, Robiscek et al. found that, in a model of attentional deficits in juvenile rats, intraprefrontal injection of exogenous active mitochondria improved cognitive performance and averted mitochondrial dysfunction (Figure 1) [112]. Although uncertainty surrounds the underlying mechanism, the presence of unbound mitochondria in cerebrospinal fluid (CSF) has been reported in animal models and subarachnoid hemorrhage patients. Despite the unknown function of these liberated mitochondria, the presence of CSF mitochondria with a higher membrane potential is a positive sign [113].

Regarding systemic or intracarotid peripheral administration, it is a less intrusive and safer approach that allows for bigger injection volumes and repeated administrations (Figure 1) [114]. The existence of the blood–brain barrier (BBB), which prevents most medications from accessing the brain, and drug diffusion in the entire body are the main barriers to intravenous injection. Finally, intranasal administration offers an alternate route for brain delivery that avoids the BBB. In this regard, Alexander et al. reported that two administrations of mesenchymal stem-cell-isolated mitochondria to rodents restored executive functioning, working memory, and spatial memory (Figure 1) [115]. Using confocal imaging, it was determined that mitochondria administered nasally were swiftly internalized by macrophages in the meninges [115]. Additionally, the administered mitochondria accessed the rostral migratory stream and various other brain regions, including the hippocampus, where they colocalized with GFAP+ cells. Myelin structural regeneration in the cingulate cortex and synaptic loss in the hippocampus were associated with the recovery of cognitive function [115]. Nevertheless, considerations such as restricted dosage volume, tiny absorption surface, the presence of degrading enzymes, and other variables related to patient congestion and mucus limit the efficacy of this route [116]. As a result, a greater knowledge of the processes driving mitochondrial delivery and cellular absorption will aid in the clinical translation of mitochondrial transplantation [94]. However, conjugation with the carrier peptide Pep-1 (a cell-penetrating peptide that has been used to facilitate the cellular uptake of nanoparticles, DNA, and proteins) has recently been demonstrated to be an effective strategy for enhancing the mitochondrial transfer [117].

## 6. Therapeutic Potential of Mitochondrial Transfer in Metabolic Disorders

The incidence and prevalence of metabolic diseases, such as obesity, diabetes, and nonalcoholic fatty liver disease (NAFLD), are increasing globally. In 2015, there were an estimated 604 million obese adults and 414 million diabetics. Diabetes prevalence is projected to reach 629 million by 2045 [118]. Approximately 85% of type 2 diabetes (T2D) patients are either overweight or obese. Recent evidence suggests that intercellular mitochondrial transfer plays a role in immune regulation [119]. Oxidative stress (OS), insulin resistance (IR), and metabolic diseases are all linked to mitochondrial dysfunction, and all three of these conditions—obesity, diabetes, and NAFLD—are on the rise [120]. Therefore, it is anticipated that the restoration of mitochondrial homeostasis will have a potential therapeutic effect on metabolic diseases and their complications [121]. Previously, Spees et al. (2006) published the evidence of mitochondrial transmission between mammalian cells [10]. However, more than a decade later, the evidence shows that mitochondrial transfer occurs between various cell types, including mMSCs and alveolar cells, astrocytes and neurons, and bone marrow mesenchymal stem cells (BM-MSCs) [122]. Additionally, the bioenergetics of injured mammals and cells may be restored via mitochondrial transfer via actin-based TNTs, extracellular vesicles (EVs), cell fusion, and cell extrusion [123]. In this regard, Court et al. found that the incorporation of exogenous mitochondria promotes the programming of regulatory T cells at a steady state, indicating that the transfer of mitochondria may have anti-inflammatory properties and consequently play a role in metabolic disorders [124]. Metabolic crosstalk between adipocytes and immune cells is necessary for tissue homeostasis and, when dysregulated, can induce pathological inflammation that leads to obesity and metabolic dysfunction associated with obesity [125,126,127]. 

MSCs have been the subject of substantial research in regenerative medicine and medication delivery because of their low immunogenicity and targeting characteristics [128]. By transferring mitochondria, BMSCs improve mitochondrial activity in hyperglycemia-induced proximal-tubule ECs (PTECs) in STZ-induced diabetic rats. When isolated mitochondria are injected directly under the renal capsule of STZ rats, the morphology of the PTECs, the structure of the tubular basement membrane, and the brush boundary all improve rapidly. The expression of megalin and sodium-glucose cotransporter-2 (SGLT2) are both increased when isolated mitochondria are administered, whereas nuclear translocation of receptor-activated receptor-coactivator-1 (PGC-1a) is decreased [72]. Interestingly, insufficient insulin secretion by islet cells leads to the metabolic condition diabetes mellitus. Insulin secretion by islet cells is dependent on glucose’s stimulation of mitochondrial ATP synthesis. To improve the insulin secretion capacity of injured beta cells, human adipose MSCs under coculture conditions were able to transfer mitochondria to human islet cells [129]. In addition, cells can maintain proper energy metabolism by either ingesting mitochondria to increase energy or discarding them. Nicolas-Ávila et al. showed new evidence that suggests that resident macrophages in the myocardium can maintain cardiac homeostasis and prevent metabolic dysfunction by absorbing and degrading faulty mitochondrial particles. Perhaps more intriguing is the fact that adipose tissue macrophages can absorb the mitochondria of neighboring fat cells [130]. Thus, this mitochondrial translocation between adipose tissue and adipose tissue macrophages (ATMs) is a mechanism of immune-metabolic interaction that controls the metabolic balance [131]. However, poor energy balance and increased susceptibility to diet-induced obesity occur when this transfer is disrupted in obese people due to a lack of heparan sulphate (HS). Therefore, the precise control of mitochondrial transport across cells might be a novel therapeutic approach for the treatment of metabolic disorders [9]. 

**Figure 2:**
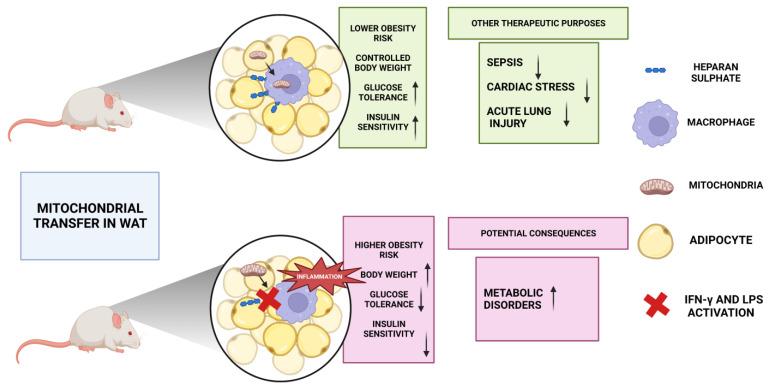
The mitochondrial exchange between adipocytes and macrophages in adipose tissue is described. Both promising treatment avenues and potential repercussions of an incorrect transfer are presented. The figure is based on Brestoff et al.’s study [131] and pictures as well as [9,133].

A recent review carried out by Valenti et al. (2021) showed that the transfer of intercellular mitochondria occurs in vivo in white adipose tissue (WAT) via an adipocyte-to-macrophage mitochondrial transfer axis that is dysregulated in obesity. Showing some studies such as Brestoff’s and colleagues, this review explained that the intake of mitochondria by white adipose tissue (WAT) macrophages is diminished under nutritional conditions of diet-induced obesity, thereby decreasing the efficiency of this process (Figure 2) [131]. First, the proinflammatory environment caused by interferon-γ (IFN-γ), lipopolysaccharide (LPS) activation, and M1 polarization impairs the mitochondrial uptake of macrophages intrinsically. It has been reported that the heparan sulphate (HS) biosynthesis pathway has anti-inflammatory effects [131]. Brestoff et al. (2021) finally found that inhibiting heparan sulphate biosynthesis results in abnormal mitochondrial uptake, decreased energy expenditure, and fat accumulation in the adipose tissue [131]. Due to this, research has demonstrated that mitochondrial transfer between cells may contribute to metabolic diseases [132]. Concretely, HS is essential for mitochondrial uptake in macrophages, as demonstrated by genome-wide clustered regularly interspaced short palindromic repeats (CRISPR)–CRISPR-associated protein 9 (Cas9) silencing [133]. Mice with obesity or deletion of the myeloid-cell HS biosynthesis gene exostosin-1 (Ext1) show lower HS levels in WAT macrophages, leading to decreased intercellular mitochondrial transfer from adipocytes to macrophages, which in turn leads to weight gain and impaired glucose tolerance and insulin sensitivity (Figure 2) [88,131]. More study is needed to determine the history of ATMs, the features of the transcriptionally different macrophage subgroups, and their reaction to the metabolic status in obesity. In addition, evidence suggests that ATMs are extremely plastic in relation to their surroundings, and obesity is strongly associated with the number, origin, and functional alterations of ATMs [134]. Uncertainties in culture method, donor cells, growth level, storage and shipping circumstances, and ethical sanction also limit MSCs’ clinical utility. Platelets are a more desirable source for autologous mitochondrial transplantation due to their availability, abundance, and minimal immunogenicity [121]. However, Pang et al. (2021) showed that therapeutic encouragement of mitochondrial transfers or mitochondrial transplantation is a viable therapy for disorders, such as heart stress, obesity, acute lung damage, and sepsis, since these conditions all include mitochondrial dysfunction (Figure 2) [74,130,135].

Regardless of the above, mitochondrial genome engineering and mitochondria–nucleus hybridization have led to the expansion of our knowledge of mitochondrial biology and its potential therapeutic applications [136]. In this regard, several different types of recipient tissues benefit from the increased functionality made possible by the transfer of mitochondria. Improved cardiac systolic performance, decreased infarcted area, and increased ATP generation have all been demonstrated after transplanting isolated mitochondria into an ischemic heart [102,137]. As mentioned earlier, in inflammatory and tissue damage settings, macrophages can boost their anti-inflammatory and phagocytic capabilities by ingesting mitochondria [74]. Kim et al. reported a study’s description of a technique for introducing mitochondria into cells. It offered novel approaches that suggested promising directions for future treatments [138]. In comparison to other methods, this methodology may be both quicker and less complicated [11,139]. These authors demonstrated that isolated mitochondria could be effectively transplanted into target cells using our straightforward centrifugation approach. These cells included mitochondrial DNA-deleted Rho0 cells and atrophic muscle cells induced by dexamethasone. The results showed that mitochondrial transfer normalized the recipient cells’ oxygen consumption rate, mitochondrial ATP generation, mitochondrial membrane potential, and mitochondrial reactive oxygen species level [138]. Muscle atrophy’s underlying AMPK/FoxO3/Atrogene pathway was also halted when undamaged mitochondria were delivered to wasting muscle tissue. This fast and easy mitochondrial transfer procedure can be utilized to treat illnesses caused by mitochondrial malfunction [138], and these findings demonstrate that centrifugation-based transfer of exogenous mitochondria into target cells is safe, effective, and may increase ATP content and improve metabolic activity [138].

Regarding renal damage in diabetic nephropathy (DN), it is mitigated by umbilical-cord MSCs through the promotion of macrophage polarization towards an anti-inflammatory phenotype. This is achieved through the activation of a pathway involving the phosphatidylinositol 3-kinase EB (PGC-1a) and the transcription factor EB (TFEB), which leads to mitochondrial biogenesis and lysosomal autophagy [71]. In relation to this, there is a rising interest in studying therapeutic techniques targeting mitochondrial dysfunction in renal ischemia–reperfusion damage (IRI) as a potential treatment for acute kidney injury, which is currently only treated with preventative strategies including fluid control and dialysis [140,141]. Researchers have developed a new treatment for IRI based on the transplanting of mitochondria [142]. This treatment involves injecting the patient with respiration-competent mitochondria extracted from nonischemic tissue to replace the native mitochondria that were destroyed by IRI. In this regard, Doulamis et al. (2020), in a clinically relevant large animal model, demonstrated that intra-arterial mitochondrial transplantation is safe and effective, enhancing renal function and decreasing renal toxicity associated with AKI [73]. Concretely, an improved myocardial function was achieved by increasing ATP content and myocardial viability following the ischemic perfusion [73,143]. As similarly reflected before, the intravenous administration of exogenous mitochondria decreased serum transaminase activity in NAFLD mice, thereby reducing lipid accumulation and oxidative injury in mice with obese livers. MRT offers distinct therapeutic potential for the treatment of NAFLD and, consequently, has a promising future in the metabolic disease therapy [144].

As already mentioned, mitochondrial transfer and mitochondrial transplantation have garnered considerable interest. The investigation of spontaneous mitochondrial transfer provides a theoretical foundation for the future treatment of diseases through the mitochondrial transplantation [133]. Even though the mechanisms of mitochondrial transfer and transplantation are not well understood at present, these processes continue to hold tremendous therapeutic potential. To understand the molecular and cellular mechanisms of mitochondrial transfer/transplantation and to demonstrate their efficacy, which will serve as the basis for future clinical trials, extensive and standard experiments must be conducted [9].

## 7. Therapeutic Potential of Mitochondrial Transfer in Cancer

Cancer is a complex disease that exhibits metabolic heterogeneity. This implies that the rates of glycolysis, tricarboxylic acid cycle (TCA), and oxidative phosphorylation (OXPHOS) can be altered to sustain the energy and biomass requirements of the tumor cells [145]. The reasons behind these metabolic alterations are multifactorial and can be attributed to various genetic mutations and modifications [146] in the tumor microenvironment, such as oxygen levels, pH, and the availability of substrates. Furthermore, neighboring cells and their interactions can also contribute to the metabolic changes observed in cancer. Overall, the metabolic flexibility of cancer cells allows them to adapt to the changing conditions within and around the tumor, enabling them to proliferate and survive [147].

### 7.1. Mechanisms of Mitochondrial Transfer

As exposed before, several mechanisms have been proposed for mitochondrial transfer, including TNTs, exosomes, and cell fusion. TNTs are formed by the extension of the plasma membrane and the cytoskeleton of the cells involved in the transfer process. They have been observed in various cell types, including neurons, immune cells, and cancer cells. The transfer of mitochondria via TNTs has been shown to rescue damaged cells and improve their metabolic function [114]. Likewise, the transfer of mitochondria via exosomes has been shown to promote cellular metabolism, reduce oxidative stress, and enhance cell survival [115]. Exosomes have been implicated in various physiological and pathological processes, including immune response, cancer, and neurodegeneration [116].

Additionally, cell fusion has been shown to play a crucial role in tissue repair and regeneration, particularly in the liver and muscle tissues [117]. However, the safety and efficacy of using cell fusion as a therapeutic strategy for mitochondrial diseases remain to be evaluated [118].

Therefore, mitochondrial transfer via TNTs, exosomes, and cell fusion represents a promising therapeutic strategy for the treatment of mitochondrial diseases and tissue repair. Among these, the most intriguing and extensively studied is its role in cancer and its resistance to cancer treatment.

### 7.2. Implications of Mitochondrial Transfer in Cancer Cells

Mitochondrial transfer in cancer cells has gained increasing attention since 2019, reaching an average of 130 publications per year, and 35 in the first third of 2023. Its interest resides in the contribution of transferred cells to the acquisition of phenotypic characteristics that enhance the aggressiveness of cancer cells. 

One of the significant findings is that the transferred mitochondria can alter the metabolic profile of recipient cells, leading to an increase in proliferation, migration, invasion, and resistance to chemotherapy [148]. Additionally, the transfer of mitochondria may also influence the stemness of cancer cells, which could potentially contribute to tumor initiation, recurrence, and therapy resistance. The acquisition of phenotypic characteristics through mitochondrial transfer can be facilitated by various genetic and epigenetic changes in the mitochondrial DNA (mtDNA) [149]. Mutations, deletions, and epigenetic modifications in mtDNA can alter the expression of genes involved in energy metabolism, redox signaling, and apoptosis, resulting in the acquisition of cancer-related traits [150]. Interestingly, gain-of-function mutations in mtDNA could potentially fade away depending on the half-life of specific mitochondrial components that would directly or indirectly depend on the nuclear DNA expression of donor cells [151].

Moreover, studies have shown that mitochondrial transfer can also result in heteroplasmy, which is the coexistence of mtDNA with different genotypes within the same cell or tissue. Heteroplasmy can persist in cancer cells for a long time, and the stability of heteroplasmy can significantly impact cellular functions and phenotypic characteristics. Therefore, understanding the kinetics of mitochondrial transfer and heteroplasmy can provide important insights into the molecular mechanisms underlying cancer progression and therapy resistance [152]. Yet, more studies examining the kinetics of mitochondrial transfer and heteroplasmy are required to elucidate these phenomena and develop novel therapeutic strategies to target mitochondrial transfer and improve cancer therapy.

### 7.3. Potential Therapeutic Applications

According to the authors, there are several strategies to inhibit mitochondrial transfer and potentially enhance the efficacy of cancer therapy [151].

One approach is to target microtubule polymerization, which is required for the formation of tunneling nanotubes (TNTs) that facilitate mitochondrial transfer. Taxanes and Vinca alkaloids, which are commonly used chemotherapeutic agents, have been shown to partially inhibit mitochondrial transfer by inhibiting microtubule polymerization [153]. Thus, these agents could be used as adjuvant treatments to prevent or reduce the transfer of mitochondria from resistant to sensitive cancer cells.

M-sec, a marker and regulator of TNT formation, has also been proposed as a targetable inhibitor of mitochondrial transfer [151]. Inhibition of M-sec expression or function could potentially block the formation of TNTs and reduce mitochondrial transfer between cancer cells. Other molecules involved in TNT formation and mitochondrial fission, such as Rho GTPases, could also be targeted for therapeutic purposes [28].

In addition to inhibiting mitochondrial transfer, promoting mitochondrial dysfunction has also been suggested as a potential therapeutic strategy [154]. By targeting the electron transport chain or oxidative phosphorylation, it may be possible to induce mitochondrial stress and apoptosis in cancer cells. Alternatively, targeting mtDNA mutations or deletions that confer resistance to chemotherapy could also be a viable approach [155].

Promoting mitochondrial dysfunction or targeting mtDNA mutations may also be a viable strategy. However, further studies are needed to validate the efficacy and safety of these approaches in clinical settings.

## 8. Impact of Mitochondrial Transfer on Cell Death Pathways

Mitochondrial transfer has been shown to modulate cell death pathways through various mechanisms. One well-established mechanism is the transfer of functional mitochondria to recipient cells with compromised mitochondrial function [156]. In conditions where mitochondrial dysfunction is implicated in cell death, such as in neurodegenerative diseases like Parkinson’s and Alzheimer’s, the mitochondrial transfer has been shown to restore mitochondrial function in recipient cells and rescue them from cell death [157]. This suggests that mitochondrial transfer can have a protective effect on cell death pathways by replenishing functional mitochondria and preventing mitochondrial-dependent cell death.

Another mechanism by which mitochondrial transfer can impact cell death pathways is through the transfer of mitochondrial mtDNA and its associated factors [158]. mtDNA contains essential genes involved in oxidative phosphorylation, and mutations in mtDNA have been associated with various diseases as previously mentioned. In this line, mitochondrial transfer can facilitate the transfer of healthy mtDNA and its associated factors to recipient cells, thereby correcting mtDNA mutations and restoring mitochondrial function [159]. This may have a significant impact on cell death pathways, as healthy mtDNA can restore normal mitochondrial function and prevent cell death.

Furthermore, recent studies have also suggested that mitochondrial transfer can modulate cell death pathways through signaling pathways [160]. Mitochondria are known to release various signaling molecules, such as reactive ROS, cytochrome c, and other pro- and anti-apoptotic factors, which can influence cell death pathways [88]. Mitochondrial transfer can alter the signaling landscape of recipient cells by introducing new signaling molecules from the transferred mitochondria or by changing the balance of pro- and anti-apoptotic factors [161]. This can result in modulation of cell death pathways and influence the fate of recipient cells as the authors suggest. 

It is worth noting that the impact of mitochondrial transfer on cell death pathways can be context-dependent and may vary depending on the specific cellular context and the type of cell death pathway involved as the authors suggest [28]. For example, while mitochondrial transfer may protect cells from apoptotic cell death, it may also promote necrosis or autophagy in certain conditions [162]. The mechanism and outcome of mitochondrial-transfer-mediated modulation of cell death pathways may also vary depending on the source and quality of the transferred mitochondria, the recipient cell type, and the physiological or pathological conditions of the cells involved [28].

Thus, there are few, but increasing in number, studies showing that both stem cell transplantation and direct mitochondrial transplantation can effectively rescue tissue damage in a mitochondrial-transfer-associated manner [28]. Despite existing barriers, such as technical challenges and ethical considerations, a better understanding of the optimal conditions for mitochondrial transfer could potentially pave the way for clinical breakthroughs in the translation of mitochondrial transfer and transplantation for therapeutic purposes. Therefore, while mitochondrial transfer holds promise as a therapeutic approach for various conditions, further research is needed to fully understand its mechanisms, optimize conditions for translation to the clinic, and overcome existing challenges. The potential for exploiting mitochondrial transfer for tissue revitalization, tumor suppression, and other therapeutic applications underscores the need for continued investigations in this field.

## 9. Mitochondrial Transfer and Drug Resistance in Cancer: A Possible Approach to New Therapies

Chemotherapy and targeted therapy are currently the mainstays of cancer treatment, but drug resistance and tumor relapse remain significant challenges. There are two main mechanisms of cancer drug resistance: intrinsic resistance, which is present before any treatment, and acquired drug resistance, which is caused by adaptive responses that enable cancer cells to survive under unfavorable conditions during drug treatment [163].

In this context, investigations have clearly shown that mitochondrial transfer occurs in hematological malignancies, such as acute myeloid leukemia, acute lymphocytic leukemia, and multiple myeloma (MM) [164,165]. Surprisingly, cancer cells can transfer mitochondria to nonmalignant cells via mitophagy, a process that removes defective mitochondria [166]. Transferring mitochondria and/or mitochondrial DNA to cancer cells significantly increases mitochondrial content and improves OXPHOS, promoting proliferation and invasion [167]. However, the transfer of mitochondria from bone marrow stromal cells was shown to protect mutant hematopoietic cells during chemotherapy [167]. Thus, mitochondrial exchange occurs preferentially between nonmalignant cells and cancer cells. Cancer cells that acquire mitochondria exhibit chemoresistance [33], suggesting that this process is a promising target in the treatment of various cancers. Regarding chemotherapy and treatments, some adaptive responses may include reduced drug uptake, increased drug efflux, ineffective induction of cell death, and compensatory activation of pro-survival signaling pathways [168]. However, tumor heterogeneity contributes to the development of a whole resistance phenotype, and cancer stem cells are often involved in drug resistance by maintaining a quiescent state to evade the cytotoxicity of drugs [169].

Concerning this process, cancer cells frequently reprogram their metabolic pathways to provide flexibility in the face of endogenous and exogenous stress, including drug administration. Metabolic reprogramming is considered a hallmark of cancer, and extensive research has been conducted on the Warburg effect, which describes the preference for glycolysis by tumors in the presence of oxygen [170]. Chemoresistance caused by metabolic plasticity is often mediated by key glycolytic factors, such as HK2, GLUT1, and PKM2 [171]. This metabolic adaptation results in the production of glycolytic intermediates that activate branching pathways such as the PPP and the stress response machinery to support nucleotide synthesis and redox homeostasis, leading to escape from apoptosis and reduction in drug entry [171]. Targeting the dynamic adaptability of metabolism has shown promise in improving the efficiency of cancer therapy.

Mitochondria play a vital role in cancer metabolism, where they have a crucial function in taking up substances from the cytoplasm to fuel essential processes such as the electron transport chain (ETC), respiration, tricarboxylic acid (TCA) cycle, fatty acid oxidation (FAO), and the synthesis of macromolecules [172]. Additionally, mitochondria possess the ability to sense and adapt to stress signals in order to ensure the survival of cells [173]. Recent research has expanded our comprehension of mitochondrial metabolic changes in cancer cells, which rely on mitochondrial dynamics encompassing fusion/fission, trafficking/transfer, and communication/retrograde signaling between different organelles [174]. These adaptive processes within mitochondria are crucial for managing the stress induced by drugs, which leads to alterations in mitochondrial metabolism and subsequently contributes to drug resistance. For instance, mitochondrial fission provides an advantage to cisplatin-resistant cells compared to their non-resistant counterparts under hypoxic conditions in ovarian cancer [175]. In melanoma, resistant subclones exhibit increased oxidative phosphorylation (OXPHOS) supported by PGC-1α, which is essential for buffering oxidative stress [176]. As a result of their critical involvement in cancer cell survival, drug resistance, and various metabolic pathways, mitochondria have garnered increasing attention as a therapeutic target for cancer treatment. The understanding of mitochondrial dynamics, function, and regulation has led to the development of mitochondria-targeting therapeutics, such as metformin and CPI-613 [177].

Therefore, metabolic plasticity is now regarded as a defining characteristic of cancer due to its prominent role in drug resistance. The acquisition of cancer drug resistance was also associated with mitochondrial transfer mediated by TNT, a finding that pertains to the central role of mitochondria in many metabolic pathways [148]. Although several authors demonstrated that mitochondrial transfer plays a crucial role in the development of drug resistance in MM, its precise mechanism is still unclear; overcoming the drug resistance it causes is also a formidable challenge. Matula et al. have shown that in primary myeloma cell cultures, the mitochondrial transfer is bidirectional between bone marrow stromal cells and myeloma cells, occurring via TNT and partial cell fusion with extreme increases under the influence of chemotherapeutic drugs, whereby survival and adenosine triphosphate levels increase, whereas mitochondrial superoxide levels decrease in myeloma cells [148,178]. In addition, these authors specified that these alterations and the increase in superoxide levels in stromal cells are proportional to the quantity of mitochondria derived from the other cell type that are incorporated and the drug concentration. Although inhibition of mitochondrial transfer between stromal and myeloma cells is limited, the supportive effect of stromal cells can be effectively neutralized by influencing tumor metabolism with an inhibitor of oxidative phosphorylation in conjunction with chemotherapeutics [178]. Suzuki et al.’s research, on the other hand, demonstrated that MM cells acquire drug resistance through contact with bone marrow microenvironment constituents such as bone marrow stromal cells. Cell-adhesion-mediated drug resistance (CAM-DR) plays a significant role in this process, and therapeutic medicines that reverse resistance mediated by CAM-DR are being developed as a result. Furthermore, fresh evidence suggests that drug resistance is dynamically produced via mitochondrial transfer between MM cells and other bone marrow microenvironment cells via TNTs, opening new treatment options. If these medicines are successful in clinical trials, we will be one step closer to making MM a curable disease [165].

In this line, there are compounds that have been investigated in clinical trials as potential therapeutics for overcoming drug resistance in cancer by targeting mitochondria. In the case of metformin is a well-known drug commonly used for the treatment of type 2 diabetes, but it has also been shown to possess anticancer properties [179]. Metformin targets mitochondria by inhibiting complex I of the electron transport chain, leading to a decrease in ATP production and an increase in AMP–AMPK activation. AMPK is a key regulator of cellular energy metabolism and has been implicated in the inhibition of cancer cell growth and survival. Metformin has been shown to inhibit cancer cell proliferation, induce cell cycle arrest, and promote apoptosis, thereby overcoming drug resistance in cancer cells [180]. Moreover, metformin has been reported to modulate mitochondrial dynamics, leading to changes in mitochondrial morphology and function and impairing the adaptability of cancer cells to stress conditions, such as drug exposure [181]. Metformin has entered phase III clinical trials in various cancer types, including breast cancer [182], pancreatic cancer [183], and prostate cancer, to evaluate its efficacy in overcoming drug resistance and improving patient outcomes [184].

In the case of CPI-613 is another compound that targets mitochondria for potential cancer therapy [177]. CPI-613 is a lipoate analog that inhibits mitochondrial enzymes involved in the tricarboxylic acid cycle, specifically pyruvate dehydrogenase and α-ketoglutarate dehydrogenase. By inhibiting these enzymes, CPI-613 disrupts mitochondrial metabolism, leading to a decrease in ATP production and an increase in ROS production, ultimately resulting in cancer cell death [185]. CPI-613 has been shown to exhibit anticancer activity in preclinical studies and has entered phase III clinical trials in various cancers, including acute myeloid leukemia [186] and pancreatic cancer [187], to evaluate its efficacy in overcoming drug resistance and improving patient outcomes. However, further clinical trials are needed to evaluate their safety and efficacy in different cancer types and patient populations. The understanding of the mechanisms by which these compounds target mitochondria and modulate mitochondrial function can provide valuable insights into the development of mitochondria-targeting approaches as a strategy to overcome drug resistance in cancer.

Additionally, repurposing of existing drugs for mitochondria targeting has shown potential in tumor therapy. Metformin would be one of them as previously explained, yet, another example is doxycycline, an antibiotic that has been shown to accumulate in mitochondria and inhibit mitochondrial protein translation [188]. Doxycycline has been repurposed as a potential mitochondria-targeting drug in the context of mitochondrial transfer for cancer therapy. Studies have demonstrated that doxycycline treatment can enhance mitochondrial transfer in cancer cells, leading to restoration of mitochondrial function and increased sensitivity to anticancer drugs. By enhancing mitochondrial function and metabolism, these drugs may help to restore normal mitochondrial function in cancer cells and sensitize them to anticancer treatments [188]. 

Sahinbegovic et al. pointed out in their studies that mitochondrial transfer appears to be executed similarly in both solid and hematological malignancies, which increases the significance of this process. In addition, it highlights the importance of the tumor microenvironment and cellular plasticity in cancer progression and drug resistance. The involvement of mitochondrial transfer may shed light on the unclear mechanisms of action of certain anticancer medications. The discovery of critical molecular players such as Miro1, connexin 43, and CD38 has already paved the way for possible therapeutic targeting, despite the fact that the entire signaling apparatus governing mitochondrial transfer is still unknown [189].

Moreover, once again, safety evaluation, overcoming physiological and biological barriers, and understanding treatment mechanisms at the molecular level are crucial for the successful application of mitochondria-targeting therapeutics. Advances in omics technology and cancer genomics are expected to contribute to the identification of metabolic vulnerabilities and rational combinations of mitochondria-targeting inhibitors with standard treatments, leading to more effective strategies for cancer therapy and drug resistance management, supporting the development of precision/personalized medicine in the future.

## 10. Mitochondrial Transfer as a Tool for Tissue Engineering and Regenerative Medicine

Mitochondrial transfer is a novel technique in tissue engineering and regenerative medicine that holds immense promise for the treatment of various diseases [29]. Mitochondria, which are the powerhouses of cells, are involved in several cellular processes, including energy production, calcium regulation, and apoptosis. The transfer of mitochondria from healthy cells to damaged cells has been shown to improve cellular function and promote tissue repair [190]. This technique has been used in various animal models and has shown potential for the treatment of several human diseases, including cardiovascular disease, neurodegenerative disorders, and infertility [77,78,191].

The use of mitochondrial transfer in tissue engineering and regenerative medicine has several advantages over traditional treatments. Traditional treatments often involve the use of drugs, surgery, or organ transplantation, which can have several side effects and complications [9]. Mitochondrial transfer, on the other hand, is a noninvasive technique that has minimal side effects and complications. Moreover, it has the potential to treat several diseases simultaneously, as mitochondrial dysfunction is a common feature of many diseases [192]. One advantage of mitochondrial transfer is its specificity. Unlike traditional treatments, mitochondrial transfer targets the root cause of the disease, which is mitochondrial dysfunction. The technique allows for the selective replacement of damaged or dysfunctional mitochondria, thus minimizing the risk of side effects and improving treatment efficacy. Mitochondrial transfer can also be used to prevent the transmission of mitochondrial DNA mutations from mother to child, which is associated with several genetic disorders [121,193].

Another advantage of mitochondrial transfer is its potential to enhance tissue repair and regeneration. Mitochondria play a crucial role in cellular metabolism and energy production, which are essential processes for tissue repair and regeneration. Mitochondrial transfer has been shown to improve cellular energy metabolism, increase cell proliferation, and enhance cell differentiation, leading to improved tissue repair and regeneration. The technique has been used successfully in animal models of cardiac and neurological diseases, demonstrating its potential in tissue engineering and regenerative medicine [9].

Recent advances in mitochondrial transfer research have also demonstrated the potential to create hybrid cells, which could lead to new applications in regenerative medicine. Hybrid cells are cells that contain both the mitochondrial DNA (mtDNA) of the donor cell and the nuclear DNA of the recipient cell. This technique has been shown to be useful in cases where mtDNA mutations are present in the recipient cells, as the healthy mtDNA from the donor cells can replace the damaged mtDNA in the recipient cells [194]. While mitochondrial transfer has shown great potential in animal models, its use in humans is still in the early stages of research. However, early clinical trials have shown promising results. It was found how mitochondrial transfer in patients with idiopathic infertility showed that the technique was safe and well-tolerated and resulted in the birth of healthy infants [195]. Further research is needed to assess the long-term safety and efficacy of mitochondrial transfer in humans.

Other authors highlighted the potential of mesenchymal stromal cell (MSC) mitochondrial transfer as a cell rescue strategy in regenerative medicine. MSC mitochondrial transfer has shown promising results in preclinical studies, demonstrating its ability to rescue cells from mitochondrial dysfunction and oxidative stress, promote tissue repair, and reduce inflammation in various disease models. The authors emphasize the importance of mitochondrial transfer in restoring mitochondrial function, a key factor in maintaining cellular health, and suggest that it has the potential for treating diseases, such as neurodegenerative disorders, cardiovascular diseases, and muscle degeneration [29]. In this line, the interaction between MSCs and inflammatory cells in the process of tissue repair and regeneration is basic. MSCs play a crucial role in regulating the inflammatory response by modulating the function of immune cells, which in turn influences the regenerative potential of the tissue. It is also important to note the role of energy metabolism in mediating the cross-talk between MSCs and inflammatory cells, with both cell types utilizing different energy sources and metabolic pathways to maintain their respective functions. Targeting energy metabolism pathways in MSCs and immune cells could be a promising strategy for optimizing the resolution of inflammation and enhancing tissue repair and regeneration [196].

The acknowledged role of MSCs in maintaining the balance of hematopoietic stem and progenitor cells in the bone marrow is widely recognized. However, as the use of MSC therapies expands, it is now evident that a crucial aspect of their ability to regenerate and attract cells involves the transfer of their mitochondria to damaged cells. Although this generous behavior can have positive effects, it can also worsen cancer progression and cause chemoresistance in solid and hematological tumors. Since the relapse after chemotherapy remains a significant challenge, there is a high demand for new therapeutic approaches that target the transfer of mitochondria and metabolic regulation by MSCs [197].

MSCs possess the ability to transfer mitochondria to damaged or injured cells, thus enhancing their viability and regenerative potential. Understanding the molecular mechanisms and regulatory factors that control mitochondrial transfer in MSCs to optimize their therapeutic potential in regenerative medicine is basic for an operative use [190]. In this line, mitochondrial transfer could be a therapeutic strategy for treating ischemic conditions in the brain and eye. Ischemia, which results from a lack of oxygen and nutrients in the affected tissue, is a major cause of damage and loss of function in these organs. In this situation, mitochondrial dysfunction plays a critical role in the pathophysiology of ischemic injury and suggests that restoring mitochondrial function through the transfer of healthy mitochondria could be an effective treatment strategy. Preclinical studies have investigated the use of mitochondrial transfer in animal models of cerebral and retinal ischemia, demonstrating promising results in terms of reducing damage and improving function [198].

## 11. Limitations and Challenges in the Field of Mitochondrial Transfer

Mitochondrial transfer has been found as a promising technique for preventing the transmission of mitochondrial disorders. However, the technique is not without its limitations and challenges. In this article, we have explored the various limitations and challenges in the field of mitochondrial transfer, including the availability of healthy donor mitochondria, technical challenges in the transfer process, ethical concerns, safety concerns, regulatory challenges, and cost.

### 11.1. Availability of Donor Mitochondria

One of the major limitations in the field of mitochondrial transfer is the availability of healthy donor mitochondria. While mitochondrial donation from a third-party donor can help prevent the transmission of mitochondrial diseases, it can be difficult to find suitable donors. It was reported that only 25% of potential donors were found to be eligible for mitochondrial donation due to strict donor criteria, such as age, health status, and genetic compatibility. Additionally, the use of mitochondrial DNA from unrelated donors raises ethical concerns and may not be accepted by all patients [199].

### 11.2. Technical Challenges

Another challenge in mitochondrial transfer is the technical aspect of the procedure. The transfer of mitochondria requires the use of specialized equipment and skilled personnel, which may not be available in all medical centers. Additionally, the transfer process itself can be challenging, as it involves the delicate manipulation of cells and requires precise timing to avoid damage to the cells. These technical challenges can result in a lower success rate and increased risk of complications [200,201].

### 11.3. Ethical Concerns

The use of third-party donor mitochondria raises ethical concerns, as it involves the manipulation of genetic material from multiple individuals. Some argue that this practice violates the principle of autonomy, as patients may not fully understand the implications of the procedure. Additionally, there is concern that mitochondrial transfer could lead to the creation of “designer babies” with enhanced traits. While these concerns are important to address, studies have shown that patients and their families are generally supportive of mitochondrial transfer [202].

### 11.4. Safety Concerns

Safety concerns are also a major limitation in the field of mitochondrial transfer. While the procedure has been shown to be safe in animal studies, there is still limited data on the long-term safety and efficacy of the procedure in humans. Potential risks include the transmission of disease, the development of new mutations, and unforeseen complications arising from the transfer process itself. These safety concerns underscore the need for rigorous testing and monitoring of patients who undergo mitochondrial transfer [203].

### 11.5. Regulatory Challenges

The regulatory landscape for mitochondrial transfer is complex and varies by country. In the United States, the FDA report recommended moving cautiously, limiting future clinical trials, but is available in the United Kingdom and some other countries. The lack of consistent regulatory guidance can create confusion and uncertainty for patients and providers alike. Additionally, the regulatory approval process can be lengthy and expensive, further limiting access to the procedure [204].

### 11.6. Cost

Finally, the cost of mitochondrial transfer is a significant limitation for many patients. The procedure is currently not covered by most insurance plans, and the cost can range from tens to hundreds of thousands of dollars. This can create a significant financial burden for patients and their families, limiting access to the procedure for those who cannot afford it.

Then, while mitochondrial transfer is a promising technique for preventing the transmission of mitochondrial diseases, it is not without its limitations and challenges. These include the availability of healthy donor mitochondria, technical challenges in the transfer process, ethical concerns, safety concerns, regulatory challenges, and cost. Addressing these limitations and challenges will be critical to expanding access to this potentially life-saving procedure.

## 12. Future Directions for the Development of Mitochondrial-Transfer-Based Therapies

Mitochondrial-transfer-based therapies hold great potential in treating a variety of mitochondrial diseases and age-related conditions [205]. These therapies involve the transfer of healthy mitochondria into cells or tissues with defective mitochondria, leading to improved energy production and cellular function. As research in this area progresses, there are several future directions that can be pursued to advance the development of these therapies.

One potential direction is the optimization of mitochondrial transfer methods. Currently, there are several methods for transferring mitochondria, including injection, fusion, and uptake of isolated mitochondria or mitochondria-containing particles [206]. However, these methods have limitations in terms of efficiency, specificity, and safety. Future research could focus on improving these methods to enhance the targeting of mitochondria to specific cell types or tissues, increase the transfer efficiency, and minimize the risk of adverse effects. Another direction is the development of mitochondrial-transfer-based therapies for a wider range of diseases beyond mitochondrial disorders. Mitochondrial dysfunction has been implicated in a variety of age-related conditions, including neurodegenerative diseases, cardiovascular diseases, and cancer [205]. By improving mitochondrial function through the transfer of healthy mitochondria, these therapies could have broad applications in the treatment of these conditions.

Furthermore, research could focus on understanding the mechanisms underlying mitochondrial transfer and its effects on cellular function. While the transfer of healthy mitochondria has been shown to improve energy production and reduce oxidative stress, the precise mechanisms involved are not fully understood. Elucidating these mechanisms could provide insights into the broader roles of mitochondrial function in cellular processes and potentially uncover new therapeutic targets [10].

Another promising direction is the use of noninvasive methods for mitochondrial transfer, such as extracellular vesicles (EVs) or exosomes. These vesicles have been shown to contain functional mitochondria and can be readily taken up by recipient cells, making them an attractive option for delivering healthy mitochondria to diseased tissues [207]. Moreover, the use of EVs or exosomes could potentially avoid some of the limitations and safety concerns associated with direct mitochondrial transfer methods. Another potential direction is the use of induced pluripotent stem cells (iPSCs) for generating healthy mitochondria for transfer. iPSCs can be derived from patient somatic cells and then differentiated into any desired cell type, including those affected by mitochondrial diseases. By deriving iPSCs from patients with mitochondrial diseases, healthy mitochondria can be generated from these cells and then transferred back into the patient’s affected tissues [208]. This approach could potentially avoid the need for mitochondrial donors and provide a patient-specific therapy.

Moreover, research could focus on developing mitochondrial-transfer-based therapies for specific diseases, such as Alzheimer’s disease, Parkinson’s disease, and other neurodegenerative disorders. Mitochondrial dysfunction has been implicated in the pathogenesis of these diseases, and improving mitochondrial function through the transfer of healthy mitochondria could potentially slow or even halt disease progression [209]. Finally, another area of research could focus on optimizing the functional properties of transferred mitochondria. For instance, research could explore the use of gene-editing techniques, such as CRISPR/Cas9, to introduce specific modifications or mutations into the transferred mitochondria to enhance their function and improve their compatibility with recipient cells [210].

Overall, the development of mitochondrial-transfer-based therapies has the potential to revolutionize the treatment of mitochondrial disorders and age-related conditions. As research in this area progresses, optimizing transfer methods, expanding therapeutic applications, and understanding underlying mechanisms will be key areas of focus. Future research could focus on developing noninvasive methods for mitochondrial transfer, using iPSCs to generate patient-specific therapies, developing disease-specific therapies, and optimizing the functional properties of transferred mitochondria.

## 13. Conclusions and Implications for the Use of Mitochondrial Transfer in Disease Diagnosis

Mitochondrial transfer technique has the potential to greatly aid in the diagnosis and treatment of a variety of diseases caused by mitochondrial dysfunction. The use of mitochondrial transfer may provide a means to restore proper mitochondrial function in affected cells and tissues, leading to improved health outcomes for patients. However, it is important to note that there are still many questions that need to be answered before mitochondrial transfer can be widely adopted in clinical practice. Further research is needed to fully understand the mechanisms underlying this technique and to evaluate its long-term safety and efficacy. Additionally, ethical considerations surrounding the use of mitochondrial transfer must be carefully considered and addressed. Overall, the use of mitochondrial transfer holds great promise for improving the diagnosis and treatment of mitochondrial diseases. However, careful consideration must be given to the potential benefits and risks of this technique before it can be fully embraced in clinical practice.

## Figures and Tables

**Table 1:** Summary of improvements demonstrated in animal studies (preclinical) in different conditions after applying mitochondrial transfer as therapy.

Preclinical Trials			
Disease or Injury Model Animal	Outcomes	Title	Author
Cardiac ischemia (White Rabbits)	The transplanted mitochondria enhanced oxygen consumption, high-energy phosphate synthesis, and the induction of cytokine mediators and proteomic pathways that are important in preserving myocardial energetics.	Transplantation of Autologously Derived Mitochondria Protects the Heart from Ischemia-Reperfusion Injury	Masuzawa et al. (2013) [65]
Middle cerebral artery occlusion (Rats)	The transplantation of mitochondria decreased brain infarct volume and reversed neurological deficits.	Muscle-Derived Autologous Mitochondrial Transplantation: A Novel Strategy for Treating Cerebral Ischemic Injury	Zhang et al. (2019) [66]
Ischemia–reperfusion injury (IRI) (Pigs)	Preischemic MT by single or serial intracoronary injections provides prophylactic myocardial protection from IRI.	Preischemic Autologous Mitochondrial Transplantation by Intracoronary Injection for Myocardial Protection	Guariento et al. (2018) [67]
Parkinson’s disease (Rats)	Allogeneic and xenogeneic transplantation of peptide-labeled mitochondria after 3 months improved the locomotive activity in the PD rats.	Allogeneic/Xenogeneic Transplantation of Peptide-Labeled Mitochondria in Parkinson’s Disease: Restoration of Mitochondria Functions and Attenuation of 6-Hydroxydopamine-Induced Neurotoxicity	Chang et al. (2016) [68]
Brain ischemic (Rats)	Significantly restored the motor performance.	Transferring Xenogenic Mitochondria Provides Neural Protection Against Ischemic Stress in Ischemic Rat Brains	Huang et al. (2016) [69]
Alzheimer’s disease (Mice)	A significantly better cognitive performance was noticed in the mitochondria-treated AD mice.	Mitochondrial Transfer Ameliorates Cognitive Deficits, Neuronal Loss, and Gliosis in Alzheimer’s Disease Mice	Nitzan et al. (2019) [70]
Diabetic nephropathy (Mice)	MSCs elicited macrophages into anti-inflammatory phenotype and ameliorated kidney injury through mitochondrial transfer.	Mitochondrial Transfer from Mesenchymal Stem Cells to Macrophages Restricts Inflammation and Alleviates Kidney Injury in Diabetic Nephropathy Mice via PGC-1α Activation.	Yuan et al. (2021) [71]
Diabetes (Rats)	Isolated Mt also inhibited nuclear translocation of PGC-1α and restored the expression of megalin and SGLT2 under high glucose condition (HG) in PTECs.	Mitochondria Transfer from Mesenchymal Stem Cells Structurally and Functionally Repairs Renal Proximal Tubular Epithelial Cells in Diabetic Nephropathy in Vivo	Konari et al. (2019) [72]
Acute renal injury (Pigs)	Mitochondrial transplantation by intra-arterial injection provides renal protection from ischemia–reperfusion injury.	Mitochondrial Transplantation by Intra-Arterial Injection for Acute Kidney Injury	Doulamis et al. (2020) [73]

**Table 2:** Summary of improvements demonstrated in human studies (clinical) in different conditions after applying mitochondrial transfer as therapy.

Clinical Trials			
Disease	Outcomes	Title	Author
Acute respiratory distress syndrome	MSCs promote an anti-inflammatory and highly phagocytic macrophage phenotype through EV-mediated mitochondrial transfer.	Mesenchymal Stromal Cells Modulate Macrophages in Clinically Relevant Lung Injury Models by Extracellular Vesicle 1281 Mitochondrial Transfer	Morrison et al. (2017) [74]
Myocardial ischemia	All five patients showed qualitative improvement in left ventricular function within days, without short-term complications.	Autologous Mitochondrial Transplantation for Dysfunction after Ischemia-Reperfusion Injury	Emani et al. (2017) [75]
Ischemia–reperfusion injury	MT was associated with successful separation from ECMO and enhanced ventricular strain in patients requiring postcardiotomy ECMO for severe refractory cardiogenic shock after IRI.	Autologous Mitochondrial Transplantation for Cardiogenic Shock in Pediatric Patients Following Ischemia-Reperfusion Injury	Gauriento et al. (2020) [76]
Infertility	Mitochondrial transfer from ovarian cells and healthy oocytes could lead to improved fertility outcome in low-quality oocytes.	Autologous Mitochondrial Microinjection: A Strategy to Improve the Oocyte Quality and Subsequent Reproductive Outcome During Aging	Mobarak et al. (2019) [77]
Infertility	Mitochondrial transfer technology is a beneficial clinical option to improve oocyte quality and the subsequent clinical results for patients with recurrent failures.	Mitochondrial Transfer into Human Oocytes Improved Embryo Quality and Clinical Outcomes in Recurrent Pregnancy Failure Cases	Morimoto et al. (2023) [78]

## Data Availability

Not applicable.
